# Smoking among troops deployed in combat areas and its association with combat exposure among navy personnel in Sri Lanka

**DOI:** 10.1186/1747-597X-7-27

**Published:** 2012-07-09

**Authors:** Varuni Asanka de Silva, Nicholas ELW Jayasekera, Raveen Hanwella

**Affiliations:** 1 Department of Psychological Medicine, Faculty of Medicine, University of Colombo, Kynsey Road, Colombo 08, Sri Lanka; 2 Director General Health Services, Sri Lanka Navy, Colombo, Sri Lanka

**Keywords:** Sri Lanka, Smoking, Military personnel, War, Combat exposure

## Abstract

**Background:**

Among military personnel alcohol consumption and binge-drinking have increased but cigarette smoking has declined in the recent past. Although there is a strong association between smoking and PTSD the association between combat exposure and smoking is not clear.

**Methods:**

This cross sectional study was carried out among representative samples of SLN Special Forces and regular forces deployed in combat areas. Both Special Forces and regular forces were selected using simple random sampling. Only personnel who had served continuously in combat areas during the one year period prior to end of combat operations were included in the study. Females were not included in the sample. The study assessed several mental health outcomes as well as alcohol use, smoking and cannabis use. Sample was classified according to smoking habits as never smokers, past smokers (those who had smoked in the past but not within the past year) and current smokers (those smoking at least one cigarette within the past 12 months).

**Results:**

Sample consisted of 259 Special Forces and 412 regular navy personnel. Prevalence of current smoking was 17.9% (95% CI 14.9-20.8). Of the sample 58.4% had never smoked and 23.7% were past smokers. Prevalence of current smoking was significantly higher among Special Forces personnel compared to regular forces. (OR 1.90 (95% CI 1.20-3.02). Personnel aged ≥35 years had the lowest prevalence of smoking (14.0%). Commissioned officers had a lower prevalence (12.1%) than non commissioned officers or other ranks. After adjustment for demographic variables and service type there was significant association between smoking and combat experiences of seeing dead or wounded [OR 1.79 (95%CI 1.08-2.9)], handling dead bodies [OR 2.47(95%CI 1.6-3.81)], coming under small arms fire [OR 2.01(95%CI 1.28-3.15)] and coming under mortar, missile and artillery fire [OR 2.02(95%CI 1.29-3.17)]. There was significant association between the number of risk events and current smoking [OR 1.22 (95%CI1.11-1.35)].

**Conclusions:**

There was significant association between current smoking and combat experiences. Current smoking was strongly associated with current alcohol use. Prevalence of current smoking was less among military personnel than in the general population. Prevalence of smoking was significantly higher among Special Forces personnel.

## Background

Prevalence of cigarette smoking has decreased among military personnel in the recent past. A prospective study of alcohol and cigarette use in the United Kingdom (UK) armed forces from 2002 to 2005 reports that alcohol consumption and binge-drinking increased but cigarette smoking declined during this period [1]*.* Data from two large UK military health studies found that prevalence of smoking among military males aged 20–49 years was slightly lower than among the general population [2]. The prevalence of smoking decreased in lower ranks between 1998 and 2004, by 5.1% in 20–24 year olds and 6.3% in 35–49 year olds . The Department of Defence Health Behaviour Survey of military personnel in the United States (US) shows that cigarette smoking declined from 1980 to 1998, significantly increased from 1998 to 2002, and has declined- since then [3].

Although there is a strong association between smoking and PTSD the association between combat exposure and smoking is not clear [4-6]. Data from the Millennium Cohort study shows military deployment is associated with smoking initiation and smoking recidivism, particularly among those with prolonged deployments, multiple deployments, or combat exposure [7]. However the UK armed forces study which evaluated combat exposure using the same questions as the current study, did not find a relationship between the number of cigarettes smoked and combat exposure [1].

It is important to identify factors which promote smoking in the military because young adults may initiate smoking after joining the military and those who start smoking during deployment may continue the behaviour [8]. Smoking is associated with poor physical health and adversely affects military fitness levels, deployment readiness, and safety [9]. The military also provides a unique occupational environment in which tobacco control policies such as banning use in public buildings and restricting access are easier to enforce.

Combat exposure is associated with higher risk of nicotine dependence [10]. The exact mechanism of how this happens is not known. It has been suggested that PTSD and substance use disorders are related due to shared alterations in stress related neurobiological pathways [11,12]. Smoking may serve to elevate blunted positive emotions that are central to the emotional numbing component of PTSD and depression [6,13]. People with increased anxiety sensitivity may believe that smoking reduces anxiety and anxiety-related sensations and perceive the prospect of quitting as a difficult task [14,15].

Because the association between combat exposure and smoking is not clear, we decided to identify patterns of smoking and its association among military personnel deployed in combat areas for prolonged periods. Our sample consisted of Special Forces and regular forces. The overall exposure to potentially traumatic events was high among both Special Forces and regular forces [16]. The evidence about smoking in military populations is mostly from studies carried out in the US and UK and this study will provide data about smoking among military personnel from a different population.

## Methods

The study methods are described in detail in a previous publication [16]. The data was collected as part of a study comparing the mental health status of Special Forces personnel with regular forces of the Sri Lanka Navy (SLN). The Sri Lanka Defense Forces were involved in a civil war for nearly 30 years and combat operations ended in 2009. Data collection commenced three months after combat operations ended.

This cross sectional study was carried out among representative samples of SLN Special Forces and regular forces deployed in combat areas. Both Special Forces and regular forces were selected using simple random sampling. The sample of SLN Special Forces was selected from the Special Boat Squadron. The sampling frames used were the lists of personnel from the navy central data base. Samples were selected using computer generated random numbers.

Only personnel who had served continuously in combat areas during the one year period prior to the end of combat operations were included in the study. Since there were no females in the Special Forces, females were excluded from the regular forces group. A total of 259 Special Forces and 412 regular navy personnel were recruited to the study.

### Outcome measures

The 28 page questionnaire used in the study “Health of UK military personnel deployed to the 2003 Iraq war” was used as the data collection instrument [17]. Permission was obtained from the authors for the use of the questionnaire. The questionnaire assessed several mental health outcomes as well as alcohol use, smoking and cannabis use. Sample was classified according to smoking habits as never smokers, past smokers (those who had smoked in the past but not within the past year) and current smokers (those smoking at least one cigarette within the past 12 months).

### Ethical approval

Ethical clearance was obtained from the Ethics Review Committee of the Faculty of Medicine, University of Colombo. Participation was voluntary and written informed consent was obtained from all participants. The questionnaire did not identify the participants by name.

### Statistical analysis

We analysed the prevalence of never smokers, past smokers and current smokers according to demographic variables. Association between smoking and combat exposure was explored using multiple logistic regression analyses which adjusted for demographic variables and service type. Mean difference in number of cigarettes smoked between groups was assessed using the *t* test and ANOVA. Because of the small number of PTSD cases, negative binomial regression analysis was done using the PCL-C score as a count variable to look for association between the number of symptoms and smoking status. The PCL-C total score was recoded to range from 0 to 68. Statistical analysis was carried out using SPSS version 13.0 for Windows. Stata version 10.0 was used for the negative binomial regression.

## Results

### Study sample

The sample consisted of 259 Special Forces and 412 regular navy personnel. The mean age of the sample was 27.6 years (SD 5.02). Three hundred and twenty nine (49.0%) were single, 333 (49.6%) were married and 2 (0.3%) were previously married. There were 33 (4.9%) commissioned officers, 104 (15.5%) non commissioned officers and 534 (79.6%) other ranks. Two hundred and thirty six (35.2%) were engaged in combat duty, 195 (29.1%) served on board naval vessels and 237 (35.3%) were engaged in noncombat duties which included medical, logistic, engineering, communication and administrative roles [16].

### Prevalence of smoking

Smoking habits according to demographic characteristics are shown in Table 1. Prevalence of current smoking was 17.9% (95% CI 14.9-20.8). Of the sample 58.4% had never smoked and 23.7% were past smokers. Prevalence of current smoking was significantly higher among Special Forces personnel compared to regular forces. (OR 1.85 (95% CI (1.16-2.94). Combat exposure was categorised based on number of risk events experienced. The significant difference in smoking between Special Forces and regular forces disappeared when we adjusted for combat exposure [OR 1.32 (95% CI 0.83-2.09)]. Personnel aged ≥35 years had the lowest prevalence of smoking (14.0%). The differences in prevalence of current smoking according to age, education, rank or marital status were not statistically significant.

**Table 1:** Smoking habits according to demographic characteristic

	**Never smokers prevalence % (95% CI)**	**Past smokers prevalence % (95% CI)**	**Current smokers prevalence % (95% CI)**	*Adjusted OR (95% CI) Wald, significance
**Service type**				Wald 6.7, df = 1, p = 0.01
Special Forces	48.6 (42.52-54.78)	27.8 (22.3-33.3)	23.6 (18.8-28.8)	1.85 (1.16-2.94)**
Regular Forces	64.6 (59.93-69.20)	21.1 (17.2-25.1)	14.3 (10.9-17.7)	1.0
**Age (years)**				Wald 0.84, df = 2, p = 0.66
18-24 years	64.1 (57.4-70.9)	18.2 (12.8-23.6)	17.7 (12.3-23.0)	1.0
25-34	55.3 (50.6-60.1)	26.2 (22.0-30.5)	18.4 (14.7-22.2)	1.17 (0.69-1.98)
≥35	62.0 (48.1-75.9)	24.0 (11.7-36.3)	14.0 (4.0-24.0)	0.86 (0.30-2.47)
**Marital Status**				Wald 0.41,df = 1, p = 0.52
Never married	61.3 (56.0-66.5)	19.8 (15.5-24.1)	18.9 (14.7-23.2)	1.0
Married/divorced	55.6 (50.3-60.9)	27.5 (22.7-32.3)	16.9 (12.9-20.9)	0.85 (0.51-1.40)
**Educational Status**				Wald 4.02,df = 2, p = 0.13
Less than GCE O’Level	51.2 (44.9-57.6)	29.1 (23.4-34.8)	19.7 (14.7-24.7)	1.0
GCE O Level	63.3 (57.7-68.9)	21.5 (16.7-26.2)	15.2 (11.1-19.4)	0.85(0.53-1.36)
GCE A Level or higher	60.9 (52.6-69.1)	18.8 (12.2-25.5)	20.3 (13.5-27.1)	1.51(0.84-2.70)
**Rank (Current)**				Wald 2.78,df = 2, p = 0.25
Commissioned Officer	72.7 (56.7-88.8)	15.2 (2.2-28.1)	12.1 (0.37-23.9)	0.45 (0.14-1.38)
Non-commissioned Officer	47.1 (37.4-56.9)	30.8 (21.8-39.8)	22.1 (14.0-30.2)	1.3 (0.68-2.5)
Other ranks	59.7 (55.6-63.9)	22.9 (19.3-26.4)	17.4 (14.2-20.6)	1.0

*Logistic regression analysis, Adjusted for service type, age, education, marital status and rank.

** p < 0.05.

### Number of cigarettes smoked according to demographic characteristics

There was no significant difference in the mean number of cigarettes smoked by current smokers between Special Forces and regular forces (t = 0.19, df = 669, p = 0.85). There was also no significant difference according to age groups (F = 1.13, df = 2, df = 668, p = 0.33), marital status (t = 0.75, df = 669, p = 0.94), education (F = 0.76,df = 2, df = 668, p = 0.47) or rank (F = 1.36, df = 2, df = 668, p = 0.257).

### Association between combat exposure and smoking

Combat exposure was assessed using the response to ten questions (Table 2). Previous studies have classified combat experiences in to two groups as “perceived risk to self” and “trauma involving others’ [18,19].

**Table 2:** Association between combat experience and smoking

**Combat exposure**	**Unadjusted OR Wald, significance**	**Adjusted OR Wald, significance
Discharged weapon in direct combat	1.86 (1.24-2.80)*	1.58 (0.95-2.64)
	Wald 9.07, p = 0.003	Wald 3.16, p = 0.08
Thought might be killed	1.32 (0.89-1.96)	1.24 (0.82-1.86)
	Wald 1.84, p = 0.18	Wald 1.05, p = 0.31
Seeing dead or wounded	1.97 (1.22-3.20)*	1.79 (1.08-2.9)*
	Wald 7.66, p = 0.006	Wald 5.13, p = 0.02
Handled bodies	2.60 (1.72-3.91)*	2.47 (1.6-3.81)*
	Wald 20.99, p < 0.001	Wald 16.76, p < 0.001
Aided wounded	1.56 (1.04-2.32)*	1.37 (0.90-2.10)
	Wald 4.72, p = 0.03	Wald 2.13, p = 0.14
Came under small arm fire	2.12 (1.42-3.16)*	2.01(1.28-3.15)*
	Wald 13.62, p < 0.001	Wald 9.19, p = 0.002
Came under mortar, missile, artillery fire	2.0 (1.33-2.98)*	2.02 (1.29-3.17)*
	Wald 11.37, p = 0.001	Wald 9.35, p = 0.002
Experienced landmine strikes	1.83 (0.75-4.5)	1.75 (0.70-4.36)
	Wald 1.76, p = 0.19	Wald 1.43, p = 0.23
Experienced hostility from civilians	0.91 (0.26-3.22)	0.85 (0.24-3.03)
	Wald 0.02, p = 0.89	Wald 0.06, p = 0.80
Involved in combat with enemy vessels	1.83 (1.22-2.74)*	1.52 (0.94-2.47)
	Wald 8.54, p = 0.003	Wald 2.91, p = 0.88

Test used -Logistic regression analysis, df = 1 *p < 0.05, **Adjusted for service type, age, education, marital status and rank.

Combat experiences of ‘thinking might be killed, discharging weapons in direct combat, coming under small arm fire, coming under mortar, missile, artillery fire, experiencing landmine strikes, experiencing hostility from civilians, being involved in combat with enemy vessels’ were classified as ‘risk to self’ events. Seeing dead or wounded, handling bodies and aiding wounded were classified as ‘trauma involving others’ events. Unadjusted odds ratios obtained from logistic regression analysis show that ‘risk to self’ events [OR 1.85 (95% CI 1.05-3.25) Wald 4.57,df = 1, p = 0.03] and ‘trauma involving others’ [OR 2.94 (95% CI 1.63-5.29) Wald 12.92, df = 1,p < 0.001] were significantly associated with current smoking. The significance remained after adjusting for age, education, marital status and rank for both ‘risk to self’ events [adjusted OR 1.91 (95% CI 1.07-3.410) Wald 4.78,df = 1, p = 0.03] and ‘trauma involving others’ [adjusted OR 3.04(95% CI 1.68-5.52) Wald 13.07, df = 1,p < 0.001].

Individual risk events too were associated with current smoking. Unadjusted odds ratios show that current smoking was significantly associated with discharging weapons in direct combat, seeing dead or wounded, handling dead bodies, aiding wounded, coming under small arms fire, coming under mortar, missile and artillery fire and combat with enemy vessels. After adjusting for age, marital status, education, rank and service type (Special Forces or regular forces) discharging weapons in direct combat, aiding wounded and combat with enemy vessels were no longer significantly associated with current smoking. The association with seeing dead or wounded, handling dead bodies, coming under small arms fire and coming under mortar, missile and artillery fire remained significant. These included both ‘perceived risk to self’ experiences and ‘risk events’.

There was significant association between the number of risk events and current smoking. [unadjusted OR 1.21 (95%CI 1.12-1.31) Wald 22.04, df = 1,p < 0.001] . This association remained even after adjustment for demographic factors and service type [adjusted OR 1.22 (95%CI 1.11-1.35) Wald 15.94,df = 1 p < 0.001].

### Association with mental health outcomes

Current smoking was strongly associated with current alcohol use [OR 7.41(95% CI 2.63-20.88) Wald 14.36, df = 1, p < 0.001]. This strong association remained even after adjusting for demographic variables [adjusted OR 6.7 (95% CI 2.35-19.12) Wald 12.63, df = 1, p < 0.001]. Despite high rates of combat exposure only 16 personnel fulfilled criteria for diagnosis of PTSD. Because the prevalence of PTSD was low negative binomial regression was conducted using the PCL-C symptom score as count data. Unadjusted incidence rate ratio (IRR) showed no significant difference in PTSD symptom count between smokers and non smokers (IRR 1.16 (95% CI 0.74-1.83). Current smoking was not significantly associated with GHQ caseness [OR 1.54 (95% CI 0.88-2.690 Wald 2.29, df = 1, p = 0.13].

## Discussion

Our study reports prevalence of current smoking of 17.9% (95% CI 14.9-20.8) among Navy personnel deployed in combat areas. Prevalence of current smoking was significantly higher among Special Forces personnel compared to regular forces. There was significant association between current smoking and combat experiences of seeing dead or wounded, handling dead bodies, coming under small arms fire and coming under mortar, missile and artillery fire. The number of potentially traumatic events exposed to was significantly associated with the risk of current smoking.

In our study the prevalence of PTSD was very low. There was no significant difference in PTSD symptom count between smokers and non smokers. However we did find that combat exposure was associated with current smoking. We found that both risk to life events as well as trauma to others events were associated with smoking. Handling bodies and seeing dead or wounded are classified as ‘trauma to others’ events and these significantly increased the risk of being a current smoker. A previous study has suggested that medical personnel who were exposed to such events had higher levels of psychological distress [20].

There is evidence of association between PTSD and cigarette smoking [21,22]. Several studies have found a relationship between exposure to traumatic events per se and cigarette smoking although the evidence is inconsistent [23-25]. Cumulative potentially traumatic event exposure, regardless of PTSD development, may confer greater risk for cigarette smoking and binge drinking [11,26,27]. We have previously reported that high rates of hazardous drinking and binge drinking were not present in this sample [28].

Smoking was more prevalent among Special Forces but the significant difference disappeared when we adjusted for combat exposure. Many of the previous studies have not examined the association between smoking and exposure to individual risk events. One of the few studies to have done so did not find a relationship between number of cigarettes smoked and combat exposure [1].

Prevalence of current smoking among Navy personnel was lower than that of the general population of Sri Lanka. Among males in the general population, prevalence of smoking was 29.9% in urban areas and 24.4% in rural areas [29,30]. When we compare age specific rates, we find that prevalence among <25 year age group among the SLN was higher than among the general population in urban areas (13.9%) or rural areas (9.6%) [29]. These youth may have commenced smoking after joining the Navy or prevalence of smoking may be higher among those who join the Navy. Because we do not have baseline data of smoking habits among recruits it is not possible to differentiate between these two factors.

Smoking rates are influenced by the price, availability and social acceptability of the habit. In the US military discontinuation of cigarettes rations, a zero-tolerance policy on using tobacco indoors and active tobacco-reduction campaigns have contributed to decline in use [9,31]. In the Sri Lanka Navy, cigarettes are not sold on military installations and smoking in public places within the camps are prohibited. These policies together with a declining trend in smoking among the general population explain the low rates of smoking among SLN personnel.

Our study had several limitations. Because it was a cross sectional causal relationships between smoking and combat exposure cannot be established. Self reports were used for estimation of smoking rates and under reporting is a possibility. We did not analyse the relationship between the number of cigarettes smoked and combat exposure. The study did not identify nicotine dependence as it only collected data on smoking habits.

## Conclusions

Similar to studies conducted among military personnel in US and UK we found that the prevalence of smoking was low among Navy personnel in Sri Lanka. We found an association between smoking and exposure to combat events and this should be explored further. Cumulative exposure to trauma may increase the risk of smoking

## Competing interests

RH and VAdeS are civilians who provide honorary clinical services to the Sri Lanka Navy. They do not receive any financial remuneration for their services. NELWJ is the Director General of Health Services in the Sri Lanka Navy. The Sri Lanka Navy did not have any role in the design, conduct of the study or preparation of manuscript.

## Author’ contributions

VAdeS and RH contributed to the design of the project, supervision of data collection, analysis of data and writing of the paper. NELWJ contributed to the design of the project and supervision of data collection. All authors have read the final draft and are in agreement with the content of the manuscript.
